# The potential role of microbiota for controlling the spread of extended-spectrum beta-lactamase-producing Enterobacteriaceae (ESBL-PE) in neonatal population

**DOI:** 10.12688/f1000research.10713.1

**Published:** 2017-07-25

**Authors:** Thibaud Delerue, Loic de Pontual, Etienne Carbonnelle, Jean-Ralph Zahar

**Affiliations:** 1 Département de Microbiologie Clinique et Unité de Contrôle et de Prévention du risque Infectieux, Groupe Hospitalier Paris Seine Saint-Denis, AP-HP, 125 rue de Stalingrad, 9300 Bobigny, France; 2 Service de pédiatrie, hôpital Jean-Verdier, Groupe Hospitalier Paris Seine Saint-Denis, Université Paris 13, AP-HP, 93140 Bondy, France; 3 IAME, UMR 1137, Université Paris 13, Sorbonne Paris Cité, France

**Keywords:** Extended Spectrum Beta-lactamase – Producing Enterobacteriaceae, ESBL-PE, neonatal infections

## Abstract

The spread of extended-spectrum beta-lactamase-producing Enterobacteriaceae (ESBL-PE) in the hospital and also the community is worrisome. Neonates particularly are exposed to the risk of ESBL-PE acquisition and, owing to the immaturity of their immune system, to a higher secondary risk of ESBL-PE-related infection. Reducing the risk of acquisition in the hospital is usually based on a bundle of measures, including screening policies at admission, improving hand hygiene compliance, and decreasing antibiotic consumption. However, recent scientific data suggest new prevention opportunities based on microbiota modifications.

## Introduction

One of the most worrisome challenges of the last few years is the spread of multidrug-resistant organisms (MDROs) in the community and the hospital. The rise of MDROs most frequently concerns Enterobacteriaceae isolates and is driven by the spread of extended-spectrum beta-lactamase-producing Enterobacteriaceae (ESBL-PE) ^1, 2^. The unfortunate spread of ESBL-producing bacteria can occur either by emerging clones or by horizontal transfer of ESBL plasmids between bacteria of the same or different species. This family of enzymes hydrolyzes the beta-lactam ring, rendering most beta-lactam antibiotics ineffective ^3^. As options for treatment are limited and due to the high risk of mortality related to inadequate antibiotic therapy, the clinical impact of ESBL-PE spreading is important. Neonates, specifically those hospitalized in neonatal intensive care units (NICUs), are at high risk of ESBL-PE acquisition and are highly susceptible to ESBL-PE infection due to the immaturity of their immune systems ^4, 5^. Indeed, ESBL colonization rates in pediatric intensive care units (PICUs) range up to 12% for *Escherichia coli* and 39% for *Klebsiella* spp. ^6^. ESBL rates as high as 60% for *Klebsiella* spp. and 75% for *E. coli* have been reported from blood cultures from infected infants in some NICUs ^7^. Also, as Enterobacteriaceae are recognized as serious pathogens in the NICU and rates of *E. coli* early-onset sepsis have increased along with infection in infants with very low birth weight, the spread of ESBL-PE presents major challenges in managing neonatal sepsis. According to the literature, two main factors seem to be associated with the risk of acquisition/transmission: antibiotic consumption and compliance to hand hygiene ^8^.

## Bacterial acquisition and route of transmission

Unlike adults, the newborn is exposed at birth and during the first weeks of life to multiple possible sources of MDRO acquisition. Recent studies ^9, 10^ suggested that bacterial acquisition may commence *in utero* long before delivery but that bacterial acquisition will continue during labor and within the first weeks of life. At birth, the intestinal tract becomes progressively colonized with potentially pathogenic isolates ^11^. Outside the hospital, sources of microbial colonization for newborns are parents and even other relatives ^12, 13^. In NICUs, MDRO acquisition will occur from health-care workers (as reservoirs or vectors) but also from parents and visitors.

Determining the route of MDRO acquisition in and outside the hospital seems to be very important to understand the best way to limit and control the risk of outbreaks. During NICU stay, infants are exposed to specific and non-specific factors that increase the risk of MDRO acquisition, including invasive procedures and the frequent use of broad-spectrum antimicrobial drugs ^7^. Also, transmission of ESBL-PE (and other MDRO isolates) may occur in the NICU via either medical staff or equipment; however, other sources of MDRO bacterial acquisition in infants could be the mother, and specific practices such as skin-to-skin care that, despite positive effects in the care of premature babies ^14^, could expose infants to a higher risk of acquisition ^13^.

## How to control the risk of transmission

Until now, most of the data have not allowed us to identify the modes of acquisition and to consider the best control policies. Indeed, the most frequent published reports regarding the risk factors of infection or colonization by ESBL-PE bacteria were from NICUs ^9, 15– 19^, and little is known about factors associated with spreading in the community. Most reports often describe single-unit outbreaks ^16, 17, 20^ and health care-related risk factors. Several risk factors have been identified, such as younger gestational age, low birth weight, length of hospital stay, invasive devices, antibiotic use, and hand hygiene compliance ^21– 24^. However, these different studies did not take into account several other risk factors.

Indeed, one of the most important risk factors neglected in several studies is related to colonization pressure. Nowadays, ESBL-PE colonization is considered an endemic situation, and investigators estimate that 5% to 70% of inhabitants of different countries are colonized ^25^. Analyzing risk factors of ESBL-PE acquisition in neonates without taking into account mother or family carriage (or both) may be the cause of major bias. Several studies suggested that the main route of transmission was the maternal-neonatal one ^11, 26– 29^. In a multivariate analysis in a first study conducted at the Charité - Universitätsmedizin Berlin hospital, Denkel and colleagues ^11^ identified ESBL-PE-positive mothers as the main risk factor of ESBL-PE acquisition in newborns, suggesting maternal-neonatal as the main source of acquisition ^27^. In a recent prospective study conducted in Israel ^28^ aiming to determine whether the route of ESBL-PE transmission to hospitalized newborns was from the mother, the authors found that mothers of 13 out of 14 positive newborns were colonized by ESBL-producing *E. coli*. However, in a cross-sectional study conducted in Tanzania ^29^, the prevalence of carriage of ESBL-PE was 25.4% in neonates, and the authors suggested that neonates acquire these strains from sources other than post-delivery women, such as transmission from the environment or relatives.

Several studies conducted in adult and pediatric populations have identified the measures needed to control the spread of ESBL-PE into the hospital during outbreaks ^30, 31^. Controlling the spread during outbreaks needs screening policies, decreasing antibiotic consumption, and improving hand hygiene. Indeed, surveillance may help to identify sources of infection and can be a powerful tool in the elimination of ESBL bacteria from the NICU, and the surveillance of maternal ESBL carriage can help in the prevention of neonatal colonization ^32, 33^. Moreover, prevention can occur through implementation of strict infection control guidelines, effective hand washing, minimization and safety in the use of invasive devices, and judicious use of antimicrobials such as third-generation cephalosporins ^34, 35^.

Also, little is known about how to control the spread in the community setting. Antibiotic courses seem to be a major risk factor related to the spread and acquisition in community onset ^36^.

## The role of microbiota in the acquisition of multidrug-resistant organisms

Recent clinical data has highlighted the protective role of the human microbiome in MDRO and specifically ESBL-PE acquisition. As is well known, the human microbiome plays an important role in protecting the host from *de novo* colonization with exogenous pathogenic bacteria ^37^. This colonization resistance is disrupted by exposure to antimicrobials and is one of the major risks for MDRO acquisition ^38^. In a prospective study conducted in a tertiary care center in Boston ^39^, the authors compared the fecal microbiota of healthy and hospitalized subjects and made special reference to those who acquired MDROs during their hospital stay. The fecal microbiota of the hospitalized patients had abnormal community composition, and *Lactobacillus* spp. was associated with lack of MDRO acquisition, consistent with a protective role. In a study that addressed the role of certain changes in the composition of the microbiota as a risk factor of ESBL-PE colonization, Gosalbes and colleagues highlighted a similar richness of taxa but a significant difference in species of four genera between carriers and non-carriers ^40^.

These two recent reports suggest the role of microbiome as a risk factor associated with a higher risk of MDRO acquisition. As is well known, the intestinal microbiome during infancy plays a major role in human health. Several factors influence the establishment of the microbiome in neonates, such as mode of delivery, antibiotic administration during pregnancy or after birth, and also type of feeding. For example, breast milk seems to influence initial bacterial colonization and can reduce the risk of intestinal inflammation ^41^ and has a major role in the prevention of colonization and late-onset sepsis ^42, 43^. Indeed, recent studies demonstrate the presence of a necrotizing enterocolitis-associated gut microbiome and the presence of *Clostridium perfringens* in the meconium ^44^.

These data make it possible to revive the protective role of certain bacterial species. Indeed, recent data suggested that *Lactobacillus plantarum* had antagonistic properties against both *Acinetobacter baumannii* and *Pseudomonas aeruginosa* strains ^45^; also, *Bifidobacterium breve* seems to have antimicrobial activity against several enteropathogenic strains ^46^. Moreover, oral administration of a mix of probiotics for 1 week to children on broad-spectrum antibiotics in a PICU decreased intestinal colonization by Candida and led to a 50% reduction in candiduria ^47^. However, further studies are needed to address the optimal probiotic organisms, dosing, timing, and duration ^48^.

In conclusion, several factors are associated with acquisition and transmission of ESBL-PE in infants, and our understanding of risk factors needs new studies, including the protective role of the microbiome, the impact of antibiotic consumption, and the role of maternal-neonatal transmission. The recently published studies suggesting a protective role of the microbiome for MDRO acquisition open a large field of investigation and could help us to better understand this phenomenon and to adapt our infection control measures.
